# The Case for Using Personally Relevant and Emotionally Stimulating Gambling Messages as a Gambling Harm-Minimisation Strategy

**DOI:** 10.1007/s11469-016-9698-7

**Published:** 2016-09-06

**Authors:** Andrew Harris, Adrian Parke, Mark D. Griffiths

**Affiliations:** 1 International Gaming Research Unit, Psychology Division, Nottingham Trent University, Burton St, Nottingham, NG1 4BU UK; 2 School of Psychology, University of Lincoln, Brayford Pool, Lincoln, LN6 7TS UK

**Keywords:** Decision-making, Emotion, Harm-minimisation, Pop-up messages, Responsible gambling

## Abstract

Emotions typically exert powerful, enduring, and often predictable influences over decision-making. However, emotion-based decision-making is seen as a mediator of impulsive and reckless gambling behaviour, where emotion may be seen as the antithesis of controlled and rational decision-making, a proposition supported by recent neuroimaging evidence. The present paper argues that the same emotional mechanisms can be used to influence a gambler to cease gambling, by focusing their emotional decision-making on positive external and personally relevant factors, such as familial impact or longer term financial factors. Emotionally stimulating messages may also have the advantage of capturing attention above and beyond traditionally responsible gambling messaging. This is important given the highly emotionally aroused states often experienced by both gamblers and problem gamblers, where attentional activation thresholds for external stimuli such as messages may be increased.

## Responsible Gambling Messaging Content

Responsible gambling messaging has continued to evolve, both in terms of mode of delivery, as well as message content. Classically, messages were delivered in a ‘static’ format, where messages were placed (i) nearby and/or adjacent to electronic gambling terminals, (ii) in leaflets at the gambling venue, or (iii) online where internet gamblers can access a separate internet page containing responsible gambling-related content (Harris and Parke 2016). Monaghan and Blaszczynski (2007) demonstrated that for messages to be better processed and their information retained, messages should be delivered in a dynamic mode of display in the form of a ‘pop-up’ message – messages that appear on-screen and interrupt play.

Later iterations of messages utilising this pop-up style of presentation have transitioned from containing informative style warning content, to content that is aimed at encouraging self-appraisal of gambling behaviour (e.g., Auer and Griffiths 2015a). In a questionnaire study, Gainsbury et al. (2015) had 667 gamblers complete a survey following a gambling session in a real world gambling venue. Gamblers that were exposed to messages that encouraged self-appraisal were recalled to a greater extent than messages with purely informative content, although both types of messages had a small impact on decreasing gambling consumption.

Self-appraisal encourages and facilitates autonomy, which is regarded as a fundamental psychological need for the maintenance of wellbeing and positive psychological functioning (Parke et al. 2014). In support of this notion, Deci and Ryan’s (1985, 2000) Self-Determination Theory argues that individuals have a fundamental need to engage in behaviour that is derived via their own value system and beliefs, rather than their behaviour being dictated by external influences. Consequently, more value is likely to be attributed to messaging that is not overly paternalistic, intrusive, and/or does not run contrary to an individual’s belief and value system.

The delivery style of responsible gambling messages has continued to evolve as a consequence of improving technology. Wohl et al. (2013) presented 72 electronic game machine (EGM) gamblers with educational animation videos of how EGMs work (or a neutral video) who then played an EGM in a virtual reality environment. All participants were asked to set a monetary limit on their play, but only half were reminded when that limit was reached. Both the animated video and pop-up limit reminder positively influenced gamblers to stay within their preset monetary limit. However, among participants who did not receive the pop-up reminder, those who watched the animation stayed within their preset monetary limits more than those who did not watch the animated video. For those who were reminded of their limit, there was no difference in limit adherence between those who watched the animated video and those who did not watch it.

Additionally, recent empirical research has examined the use of normative feedback as a way of promoting controlled gambling behaviour and gambling cessation, with some positive results demonstrated in samples containing high-intensity gamblers (Auer and Griffiths 2015b). However, while recent reviews of the responsible gambling messaging literature (e.g., Monaghan 2008; Harris and Griffiths 2016) highlight that while self-appraisal messaging works above and beyond informative (non-appraisal) style messaging, and that new approaches such as the use of normative feedback show promise, effect sizes and the number of gamblers being positively influenced by such messages remain small (Auer and Griffiths 2015a, b; Auer et al. 2014).

Consequently, researchers should not remain content with current approaches, and should push for new and innovative ways to positively impact upon a larger proportion of gamblers at all points along the gambling continuum from recreational and at-risk gambling through to problem and pathological gambling. The present paper argues that the use of emotionally stimulating message content designed to facilitate responsible gambling has been overlooked, or at least not given the academic attention it may warrant, particularly given theoretical and empirical accounts highlighting the important role that emotion plays in the decision-making process, as well as empirical research evidence from other potentially hazardous consumptive behaviours (e.g., smoking nicotine, drinking alcohol).

## Role of Emotion in Decision-Making

Many research studies have demonstrated that emotions constitute powerful and predictable influences over decision-making processes (for a recent review, see Lerner et al. 2015). The view of decision-making in academic literature has changed, from one negating the role of emotions, where the focus has classically been on understanding the cognitive processes involved (Lerner et al. 2015), to a view among many psychologists that emotions are in fact the dominant driver of the majority of important decisions in life (e.g., Ekman 2007; Gilbert 2006; Keltner and Lerner 2010). Indeed, this is in line with the dominant functional or adaptation approach pioneered by Darwin, where emotion is seen as a reaction to significant events that prepares action readiness and different types of alternative, possibly conflicting, action tendencies (see Darwin et al. 1998).

Indeed, decision-making and emotions may go hand-in-hand, a notion consistent with modern dual-system models of decision-making (for example, see Sohn et al. 2015). Such models postulate both an automatic, fast decision-making system (system one), as well as a more deliberate and slow decision-making system (system two; Kahneman 2011). Factors that make system one’s processes more dominant in decision-making include cognitive busyness, distraction, time pressure, and more intense mood-states, while system two’s processes tend to be enhanced when the decision involves an important object, has heightened personal relevance, and when the decision-maker is held accountable by others (Kahneman 2011).

The use of emotion in the decision-making process has often been viewed as the antithesis of controlled and rational decision-making, with arguably the majority view in Western cultures seeing emotion as having a negative impact in the decision-making process (Keltner and Lerner 2010). However, a minority of scholars, including Hume (1978), have put forward argument to the contrary, stating that reason itself should be slave to the emotions. In other words, reason should be there to modulate a positive subjective emotional state within humans, and subjective emotion itself should be the motivation and purpose of decisions. In line with this view, anger for example, provides the motivation to respond to injustice (Solomon 1993). Important in a gambling context, anticipation of regret may provide motivation to avoid excessive risk-taking (Loomes & Sugden 1982) and loss-chasing behaviour.

However, recent fMRI evidence suggests that emotionally charged states *are* associated with increased impulsive decision-making. Sohn et al. (2015) for example, demonstrated that increased impulsive decision-making is related to highly emotionally aroused states at both ends of the valence scale, with decreased activation found in prefrontal-parietal brain regions believed to be responsible for human decision-making processes associated with the deliberate system, as is suggested by dual-system perspectives on cognition (Sohn et al. 2015). These findings indicate that people tend to make impulsive decisions during emotionally aroused states compared to states of low-arousal. This is because the deliberative system’s ability to exercise regulatory control over impulsive behaviour becomes functional when individuals are not emotionally excited (Peters et al. 2006).

While it may therefore seem counterintuitive to utilise messages with heightened emotional content, it is argued that – in line with Kahneman’s (2011) approach – if emotional messages are made personally relevant, then this should encourage greater activation of the more deliberate and controlled system two approach to decision making, which may be more adaptive for the gambler. Such examples may include images of one’s family as a reminder of the consequences of loss chasing, or to frame it in a positive light, the images may encourage positive play or gambling session cessation.

## Attention Capture by Emotional Stimuli

Emotional stimuli are often shown to capture attention above and beyond neutral stimuli (Compton 2003; Vuilleumier 2005), a process that has been considered to be pre-attentive and resource independent (Ohman et al. 2001, 2001). Several research paradigms, including visual search tasks, have demonstrated preferential processing for emotional stimuli, especially when these are threat-related (e.g., Huang et al. 2008). Emotionally stimulating words have consistently been shown to produce greater response interference compared to neutral words in Stroop tasks (Compton et al. 2003; Dresler et al. 2009), hypothesised to be due to the automatic allocation of attentional resources to emotionally-laden stimuli.

A review of the literature assessing the interface between emotion and attention suggests that emotion enhances attentional processing via a two-stage process, where initially, emotional significance is evaluated pre-attentively by a sub-cortical circuit involving the amygdala; and second, stimuli deemed emotionally significant are given priority in the competition for access to selective attention (Compton 2003). Such evidence suggests that the use of emotionally stimulating messages in a gambling context may be effective as a harm-minimisation strategy on two counts. First, during a gambling session where large amounts of attentional resources are allocated to the task of gambling itself, often resulting in gamblers experiencing states of dissociation (Diskin and Hodgins 1999), emotionally stimulating messages are more likely to capture the limited attention available from gamblers, as emotional content may require less attentional effort. Secondly, emotive messaging, especially those of personal significance, may be more likely to activate selective attention through well-rehearsed schemas or neural networks, and thus enable them to have greater influence in the decision-making process.

## The Role of Positive and Negative Emotion in Responsible Drinking, and Anti-Smoking Campaigns

While strong empirical evidence exists demonstrating the effectiveness of tobacco control media campaigns in encouraging smoking cessation (Sims et al. 2014), only more recently has attention been directed towards the impact of different emotional messages and how these can impact upon smoking behaviour measures, including smoking prevalence and cigarette consumption. Sims et al. (2014) conducted a population level observational study in the UK of smoking behaviour following both positive and negative emotive messages in televised advertisement campaigns. Positive emotive messages focused on the reasons for quitting and how to quit, whereas almost all of the negative emotive messages focussed on the health risk of smoking. Both positive and negative emotive messages delivered via televised advertising were associated with a reduction in smoking prevalence when compared to the effects of emotionally neutral messages, whereas only negative emotive ads were associated with a reduction in cigarette consumption among smokers, controlling for extraneous variables such as price of cigarettes.

However, the use of negative emotion-eliciting message campaigns amongst youth populations show that fear appeals are ineffective in youth alcohol, tobacco, and other drug (ATOD) prevention (Prevention First 2008). Excessive use of fear in emotive messaging may cause an individual to ignore or not believe the message as they may feel negative consequences will happen regardless of what action they take (Prevention First 2008). Even worse, fear may invoke the opposite behaviour intended by the message if the individual likes taking risks (e.g., Steele and Southwick 1981; Zimmerman 1997), which may be especially true among youth populations given the prematurity in brain regions associated with impulse control (Blakemore and Choudhury 2006).

Prevention First (2008) have suggested that as a potentially more successful approach in combating youth ATOD use, low-fear messages from credible sources that are based on facts, tied to the present, and appeal to more positive emotions should be used instead. While both fear-based messages and fact-based drug education can increase knowledge and negative attitudes toward substance use, these approaches have not been shown to reduce or prevent substance use behaviour in youth populations.

Similarly, Agrawal and Duhachek (2010) demonstrated that the use of messages stimulating negative emotion directed at the self, had perverse effects on drinking intentions among a sample of students. In relation to ‘irresponsible’ drinking, Agrawal and Duhachek’s (2010) studies show that when emotions eliciting uncomfortable perception of oneself are further stimulated in ways which threaten to heighten this discomfort, viewers of the emotional message tend to convince themselves that the message does not apply to them, in a process the authors described as ‘defensive’ processing. This results in leaving individuals more free to do what the message warned against than if it had never been received. In particular, shame-laden consumers exposed to messages which asserted that drinking might lead to additional shame-inducing situations, believed that their own drinking would not lead to those consequences, with similar findings for emotions of guilt. Conversely, messages that elicited no threat to the self but asked participants to think about the behaviour of others had the intended effects. One of the proposed mechanisms for this effect offered by Agrawal and Duhachek (2010) is that messages that overly stimulate negative emotion may cause viewers to shut down and not process the message as a form of self-protection. As an alternative, they suggested that following a warning, messages should end on a more positive note, relieving the negative emotion and defensiveness towards the message.

This is empirically supported in a Spanish study conducted by Carrera et al. (2010) investigating the impact of emotionally stimulating messages on binge drinking intentions. They found that among a student sample, a mixed message containing both negative and positive emotional content generates lower post-message discomfort than an exclusively negative message. In addition, participants experiencing the mixed emotion message reported a lower probability of performing the risk behaviour (binge drinking) in the future. The mixed message also resulted in participants being more motivated to control the danger associated with binge drinking, and showed a greater response efficacy in the mixed message condition compared to the negative only condition.

However, messages eliciting negative emotion have been shown to enhance responsible drinking efficacy. Hendriks et al. (2014) investigated whether emotions induced by anti-alcohol messages influences conversational valence about alcohol and subsequent persuasion outcomes. The study found that fear was most induced by ‘disgusting’ messages, and in turn, fear induced a negative conversational valence that elicited healthier binge drinking attitudes, subjective norms, perceived behavioural control, intentions, and behaviour. Consequently, it was suggested that health campaign messages should aim to stimulate healthy conversational valence.

Duhachek et al. (2012) highlight the fact that negative emotion is not a single construct, and investigated how feelings of shame and guilt interact with the emotional framing of a message to persuade viewers to drink responsibly. They argued that guilt is an emotion associated with a problem-focus that emphasizes the regaining of benefits, and that shame is an emotion associated with an emotion-focus that emphasizes consequences to be avoided. Duhachek et al. (2012) hypothesised that when the coping strategy activated by the emotion matches the message frame, the resulting appeal is easier for consumers to process and therefore the message would be more persuasive.

In line with their predictions, results of their study showed that guilt appeals are more persuasive when combined with messages framing benefits to be gained by drinking responsibly, whereas shame appeals are more persuasive when combined with messages framing consequences to be avoided. They show that guilt/benefit framed messages and shame/consequence framed messages reduce intentions to binge drink, as well as reduce the willingness to view alcohol-related advertising. The authors claimed that these messages are effective because they facilitate the use of coping strategies associated with guilt and shame.

## Positive Framing of Messages

While some evidence exists for the use of negative valence messages in the prevention of hazardous behaviours, particularly when considering the multidimensional nature of negative emotion, research seems to highlight the need for positive framing of messages, particularly among younger adults. Particular emotional states also have the added influence of not only shaping content of thought, but can also shape the depth of processing of information. Negative affect can reduce the accuracy of thin-slice judgements (a term used to mean taking quick decisions on the basis of limited information; Ambady and Gray 2002). For example, those participants experiencing negative affect in a study by Ambady and Gray (2002) showed reduced accuracy in thin-slice judgments of teacher effectiveness, except when those participants experienced cognitive load, which suggests this decrease in accuracy was caused by more deliberative processing strategies.

This is of particular relevance in a gambling context, where pop-up messages are typically delivered to players when they have reached pre-set monetary (or time) limits (e.g., Stewart and Wohl 2013). If gamblers are then informed by a pop-up message that they have spent their limit and asked if they would like to continue play, it is argued this decision will be impaired by the negative affective state caused by the monetary loss. Consequently, the use of emotionally stimulating messages should frame gambling cessation in a positive way. For example, rather than a focus on monetary loss, a message might focus on protection of money yet to be spent, for example, *“save the rest of your money for that family trip next month”*. In their study of responsible gambling strategies comparing those that gambled responsibly (so called ‘positive play’ gamblers) and problem gamblers, Wood and Griffiths (2015) suggested that this ‘carrot versus stick approach’ may be an effective way to actively promote positive play amongst all gamblers, rather than, or in addition to, more traditional ‘stick-based approaches’ that may focus on negative impacts of gambling in a paternalistic way.

This strategy is in line with ‘reappraisal’ approaches to emotional control, where reappraisal has consistently emerged as a superior strategy for dissipating an emotional response (Gross 2002). As well as reducing self-reported negative feelings in response to negative events, reappraisal has been shown to reduce both physiological and neural responses to those events (e.g., Jamieson et al. 2012; Ochsner et al. 2002), which overall may reduce the likelihood of a gambler impulsively choosing to continue play despite likely negative consequences.

## Emotionally Stimulating Messages in a Gambling Context

In support of the proposition made in the present paper, one empirical study has touched upon the idea of using graphic messages to elicit an emotional response in a gambling context. Munoz et al. (2014) assessed the impact of graphic warning messages (in combination with text-based messages) versus text-only messages, in terms of their impact on gamblers’ levels of processing of the message, cognitive appraisal, fear, and attitudes. The graphic warning message was a picture of an electronic gaming machine (EGM) being depicted as a monster eating a gambler, designed to invoke fear. The image also contained smaller embedded graphics within the EGM monster that depicted the negative (financial or family) outcomes that gamblers might experience due to excessive gambling.

Their results indicated that the presence of a graphic enhanced both cognitive appraisal and fear, as well as having positive effects on the depth of information processing. In addition, graphic content, combined with text focused on family disruptions, was more effective in changing attitudes and complying with the warning compared with other combinations of the manipulated variables (i.e. graphic versus non-graphic, and family focus versus financial focus text). However, Gainsbury et al. (2015) found that messages that specifically focus on money spent during gambling had the biggest impact in reducing gambling consumption when compared to other forms of self-appraisal messages and informative messages.

Despite these preliminary findings, the present paper argues that while the use of emotional content in a responsible gambling message context may be able to create an orientating attentional, and ultimately, a greater behavioural effect, this is likely to be more effective for stimuli that is seen as both personally relevant *as well as* emotionally stimulating. Evidence has shown that some gamblers carry specific items with them when they gamble (Griffiths and Bingham 2005). While some may argue this is more of an irrational superstitious or ritualistic behaviour with no place in science, specific items (such as photos of their family) may be used as a tool to help one make sensible decisions by reminding them of what is at stake and the negative consequences of gambling. In addition, such photos may exert an enhancing cognitive effect, allowing players to perform better by providing a positive affective state, where positive affect in most circumstances enhances problem solving and decision making, leading to cognitive processing that is not only flexible, innovative, and creative, but also thorough and efficient in a wide array of contexts (Isen 2001).

Allowing gamblers to self-create and set their own messages before gambling, while in a state of non-emotional arousal, also means they can use cues that potentially have a calming influence. For example, a gambler may choose to use a message (or cue) that reminds them of a positive event, place, or ‘thing’, which may be able to assist a transition from a state of highly aroused ‘hot’ emotion, to ‘cold’ emotion, where gambling-related decisions are more likely to be carefully deliberated (see Ladouceur et al. 2012).

## Summary and Recommendations

As noted above, empirical evidence from research examining prevention messaging on other potentially addictive behaviours demonstrates a potential asymmetric effect of fear-based messages relating to the age of the participants, where fear-based campaigns appear to be less effective for younger adults. In addition, a focus on family may be less effective for younger adults, or indeed gamblers with no dependants or those who are not in a relationship irrespective of age. This is an argument allowing gamblers to self-set the messages they receive, as they are best positioned to determine what would invoke an emotional response and motivate them to avoid excessive gambling, be it fear-based messages, or a more positively-valenced approach. Allowing gamblers to self-set the messages would require account-based play, more typical of online gambling sites, or jurisdictions where player cards are a mandatory requirement such as Norway and Sweden.

Emotional messages could be delivered via text and/or images. Empirical studies need to ascertain which mode of presentation is more effective, or indeed if there is an additive effect of having both. This may be less relevant for online modes of play, or jurisdictions with mandatory account-based play, as gamblers can set the mode of message most effective and preferred by them, which is likely to differ on an individual-by-individual basis.

While it is generally accepted that harm-minimisation tools are best used as a preventative measure to help non-problem or at-risk gamblers stay in control, as opposed to an intervention measure for problem gamblers, this should not be assumed. Indeed, the testing of emotionally stimulating messages should include gamblers across the entire playing continuum, ranging from part-time recreational gamblers through to pathological gamblers. In addition, current messaging approaches used as a harm-minimisation strategy should be tested in conjunction with the use of emotional messaging to test for potential additive benefits. It is proposed that self-appraisal messages, that demonstrate responsible gambling efficacy, should follow emotionally stimulating messages, as emotional messages are likely to create a greater orientating response which may then result in subsequent messages receiving greater attention, having a deeper level of processing, and ultimately, having a greater behavioural impact.

With any new approach to harm-minimisation, there are potential unintended consequences on behaviour, highlighting the need for new approaches to be first tested in controlled environments before being rolled out at population level. With the use of emotionally stimulating messaging, it is important to ensure that a focus on financial or familial consequences of excessive gambling does not have the unintended effect of promoting loss-chasing behaviour. For example, if gamblers view that they have passed a significant threshold of loss, quitting the game may not be an option for them as they may see any further loss as insignificant. Presenting financial or familial messages may only further compound this view.
